# Hybrid *cis*-stilbene Molecules: Novel Anticancer Agents

**DOI:** 10.3390/ijms20061300

**Published:** 2019-03-14

**Authors:** Natalia Piekuś-Słomka, Renata Mikstacka, Joanna Ronowicz, Stanisław Sobiak

**Affiliations:** Department of Inorganic and Analytical Chemistry, Nicolaus Copernicus University in Toruń, Ludwik Rydygier Collegium Medicum in Bydgoszcz, 85-089 Bydgoszcz, Poland; natalia.piekus@cm.umk.pl (N.P-S.); joanna.ronowicz@cm.umk.pl (J.R.); ssobiak@cm.umk.pl (S.S.)

**Keywords:** hybrids, stilbenes, anticancer agents

## Abstract

The growing interest in anticancer hybrids in the last few years has resulted in a great number of reports on hybrid design, synthesis and bioevaluation. Many novel multi-target-directed drug candidates were synthesized, and their biological activities were evaluated. For the design of anticancer hybrid compounds, the molecules of stilbenes, aromatic quinones, and heterocycles (benzimidazole, imidazole, pyrimidine, pyridine, pyrazole, quinoline, quinazoline) were applied. A distinct group of hybrids comprises the molecules built with natural compounds: Resveratrol, curcumin, coumarin, and oleanolic acid. In this review, we present the studies on bioactive hybrid molecules of a well-known tubulin polymerization inhibitor, combretastatin A-4 and its analogs with other pharmacologically active entities. The mechanism of anticancer activity of selected hybrids is discussed considering the structure-activity relationship.

## 1. Introduction

A great number of diseases, such as cancer, cardiovascular disease, diabetes, and neurodegenerative diseases, are caused by a combination of genetic and environmental factors. The therapy of multifactorial disorders is directed at several targets responsible for the disease. Consequently, in complex diseases, therapeutic strategies are based on the use of multi-target agents. The aim of a new multi-targeted approach is to enhance the efficacy of therapeutic agents by directing them towards more than one target, and concomitantly to improve their bioavailability and to avoid multi-drug resistance. The design and synthesis of molecules that are hybrids of different bioactive compounds bound together by covalent bonds ensure the achievement of the multi-directional therapeutic effect. The innovative approach of hybrid design is to act with a single molecule on several biological targets with a high probability of better efficacy due to an additive or synergistic effect [1,2].

In the design of new antitubulin agents, the problem of tumor resistance, due in particular to transporter P-glycoprotein (Pgp) engaged in drug efflux, should be addressed. In cases of anticancer agents targeting tubulin, mutations in the tubulin subunits, changes in the tubulin isotype composition of microtubules and altered expression or binding of microtubule-regulatory proteins have to be considered as factors influencing drug resistance [3]. To deal with mutations contributing to drug resistance, the preparation of hybrid compounds with entities of different mechanism of action seems to be more effective than using a single agent.

The growing interest in anticancer hybrids in the last few years has resulted in a great number of reports on hybrid design and synthesis [4,5,6,7]. The search for more potent anticancer agents was focused on compounds which could overcome the drug resistance of cancer cells. Some novel multi-target-directed drug candidates were synthesized, and in preclinical studies, their anticancer potential was evaluated. In the design of hybrid molecules, two approaches can be observed; the first is based on the link of two or more bioactive moieties with covalent bonds, and the second is based on the ring geometry when the pharmacophores form the molecule with the scaffold resembling that of the known anticancer agent [8]. For the design of hybrids showing enhanced anticancer activity, the molecules of pyrazole, imidazole, triazole, pyridine, pyrimidine, benzimidazole, quinazoline, quinone, quinolone, and isatine were applied. A distinct group of hybrids comprises the combined molecules built with such natural compounds as resveratrol [9,10], curcumin [11,12], coumarin [13], and oleanolic acid [14]. Further, the design of hybrids consisting of two known therapeutics is a new approach in drug development introduced in studies of Erlotinib-NSAID (nonsteroidal anti-inflammatory drugs) hybrids [15]. This review focuses on hybrid molecules obtained with the combination of combretastatin pharmacophore with other bioactive entities. The hybrids of *iso*statin and *iso*combretastatin are also included. The biological properties of synthesized conjugates with regard to structure–activity relationship are presented and discussed to highlight the perspectives of these hybrids in anticancer drug development.

## 2. Combretastatins: Structure and Activity

**Figure 1:**
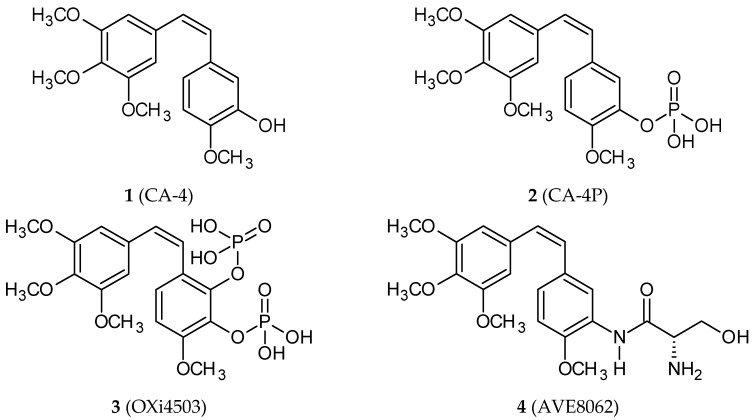
Chemical structures of combretastatin A-4 (**1**) and its analogs in clinical trials (**3**–**4**).

Combretastatins are a class of natural compounds isolated from the African bush willow *Combretum caffrum* in the 1970s [16]. They are *cis*-stilbene (1,2-diphenyl-*cis*-ethene) derivatives. The best-known combretastatin A-4 (CA-4; 3,4,5,4′-tetramethoxy-3′-hydroxy-*cis*-stilbene; **1**) (Figure 1) displays cytotoxic activity against numerous cancer cell lines [17]. CA-4 and its phosphate, which is a more soluble form of CA-4, interact with tubulin, inhibiting its polymerization, damaging epithelial cells and exhibiting antivascular activity [18]. Since the discovery of CA-4′s promising bioactivity, studies of its properties and bioactivities of its analogs have been conducted with the purpose of finding compounds characterized by more potent and specific activity and better pharmacological characteristics [19]. Most synthetic derivatives possess the trimethoxyphenyl group linked to substituted aromatic moieties through two/three-atom bridges or heterocyclic rings, including oxazoles and imidazoles. In 2003, the phosphate of CA-4 (CA-4P; fosbretabulin; Zybrestat™; **2**) (Figure 1) was approved by both the FDA and European Medicines Agency as an orphan drug for the treatment of anaplastic thyroid cancer. Many of CA-4 analogs are in preclinical studies as potential anticancer agents; three of them (fosbretabulin; OXi4503, **3**; AVE8062, **4**) (Figure 1) are in clinical trials of combined therapy with paclitaxel. 

Tubulin is the main target on which combretastatins act. Heterodimers of α- and β-tubulins polymerize to form microtubules that are major components of the cytoskeleton, playing important roles in structural support, intracellular transport, and DNA segregation. At the beginning of mitosis, the interphase microtubular network forms a mitotic spindle that segregates the replicated chromosomes into daughter cells. Microtubule interfering agents (MIAs), including anti-cancer drugs, cause suppression of microtubule dynamics either by inhibiting tubulin polymerization (e.g., colchicine, vinblastine, vincristine, vinorelbine) or by stabilizing microtubules (e.g., paclitaxel, docetaxel). Both effects result in G2/M arrest of the cell cycle and cell death [20,21,22]. MIAs exhibit affinities with specific binding sites on tubulin. Colchicine binds to a site at the interface between α and β subunits of tubulin dimers, while paclitaxel, vinca alkaloids, and laulimalide/peloruside bind to the distinct sites at β-subunit, the taxoid site, vinca site, and laulimalide/peloruside sites [23]. The exact location of these sites and the mechanism of MIA interactions with tubulin is still a subject of studies. Many natural MIAs have been identified, and still, new classes of potential MIAs are synthesized; among them, potent antitubulin agents are selected [24,25,26,27]. Combretastatins which show an affinity with the colchicine binding site draw our attention in the context of the search for potent remedies against cancer. They are vascular disrupting agents (VDAs), acting against tumor neovascularization; this action results in a reduction in tumor blood flow, tumor hypoxia, and ischemic necrosis [18].

## 3. *cis*-Stilbenoid-Related Hybrids and Their Anticancer Activities

### 3.1. Combretastatin A-4 Based Hybrids

**Figure 2:**
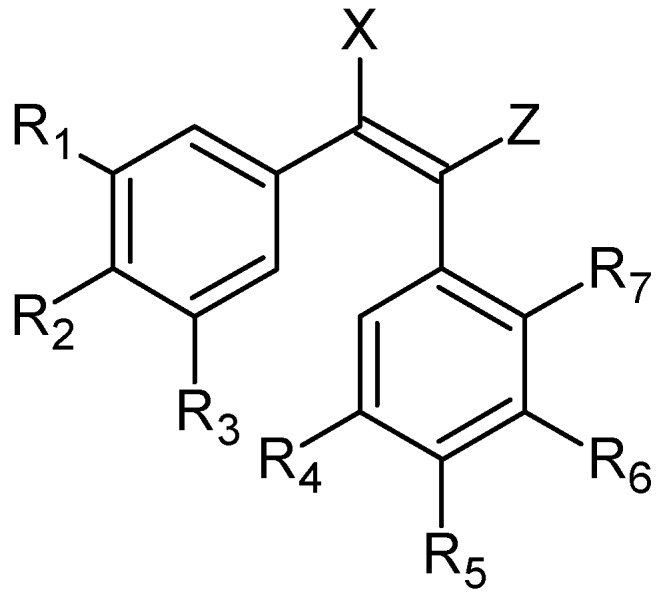
The general structure of combretastatin based hybrids.

The general structure of compounds described in this section is presented in Figure 2. Hybrid molecules were obtained through modifications in the B-ring or changes in the ethene bridge.

#### 3.1.1. Piperlongumine Hybrids

**Figure 3:**
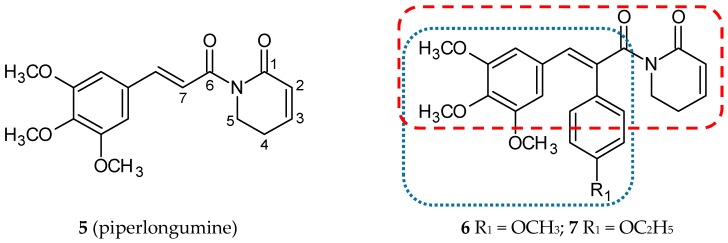
Chemical structures of piperlongumine (**5**) and its hybrids (**6**,**7**). Entity I (*cis*-stilbene) and entity II were depicted with the blue (**· · · · · · ·**), and red (**– – – –**) dotted lines, respectively.

Piperlongumine (5,6-dihydro-1-[(2-*trans*)-1-oxo-3-(3,4,5-trimethoxyphenyl)-2-propenyl]-2(1*H*)-pyridinone; **5**) (Figure 3), also called piplartine, is a compound widespread in all plants of the Piperaceae family, mainly in long pepper (*Piper longum* L.), which has been extensively used in Indian and Chinese traditional medicine. Piperlongumine was first isolated in 1961 by Atal and Banga, and its structure was correctly characterized in 1968 [28]. Nowadays, it is increasingly recognized that this amino alkaloid may be used as an agent with several properties, including geno and cytotoxic, antiangiogenic, antiplatelet aggregation, anxiolytic, antidepressant, antimetastatic, antinociceptive, antidiabetic, anti-atherosclerotic, antibacterial, antifungal, antiparasitic and, most crucially, highly cytotoxic ones against cancer cells of many different histotypes. The molecular and cellular mechanism of antitumor action has been broadly investigated [28]. An increased generation of reactive oxygen species (ROS) is one of the most promising mechanisms resulting in cell cycle arrest and apoptosis [29]. It is well known that mutations in the gene encoding p53 protein are a common cause of carcinogenesis. Many research efforts have been devoted to finding and describing small-molecules as mutant p53 reactivators [30]. On the grounds that many of these compounds possess highly electrophilic unsaturated bonds, they can be engaged in inducing oxidative stress and redox imbalance in cells.

Piperlongumine possesses two double bonds (C2-C3 and C7-C8), and moreover, its derivatives substituted at C-7 position with phenyl ring mimic the structure of CA-4. Punganuru et al. synthesized and characterized six novel C-7 piperlongumine hybrids substituted in the *para* position of C7 phenyl ring with the methoxy and the ethoxy groups, respectively [31]. Antiproliferative activities against cell lines A-549 (human lung carcinoma cell line), MCF-7 (human breast adenocarcinoma cell line), SKBR3 (human breast adenocarcinoma cell line), HT-29 (human colorectal adenocarcinoma cell line), SF188 (paediatric glioblastoma cell line), GBM10 (human glioblastoma cell line), T98G (human glioblastoma cell line) were appraised for all of the conjugates of CA-4 with piperlongumine derivatives. All of the described conjugates demonstrated a better cytotoxic activity in comparison with a parent compound (**5**). Two of them (**6** and **7**) (Figure 3) showed IC_50_ values below 1.5 µM against all tested cell lines, excluding MCF-7 and A-549. The mechanism of cytotoxicity was determined using biological assays only for the most promising structure **6**. Treatment of SKBR3 with **6** for 3 h led to a rise in ROS levels. Fluorescence microscopy and Western blot analysis proved the increase in protein glutathionylation (a marker of oxidative stress) in SKBR3 cells treated with **6**. The effect of **6** on tubulin polymerization in vitro and directly in SKBR3 cells was also investigated. Results of these tests confirmed a significant anti-microtubule activity of **6**. Moreover, flow cytometry analysis revealed that, in cells treated with **6**, cell cycle progression was arrested in the G2/M phase. All of the six derivatives were more active against the SKBR3 cells than against MCF-7 cells. Both of these cell lines come from breast tumors, but only in SKBR3 cells is the p53-R175H mutation expressed. Further studies revealed a reactivation of biological functions of the p53 mutant gene in SKBR3 cells treated with **6** [31]. 

#### 3.1.2. Estrogen Receptor Modulators

**Figure 4:**
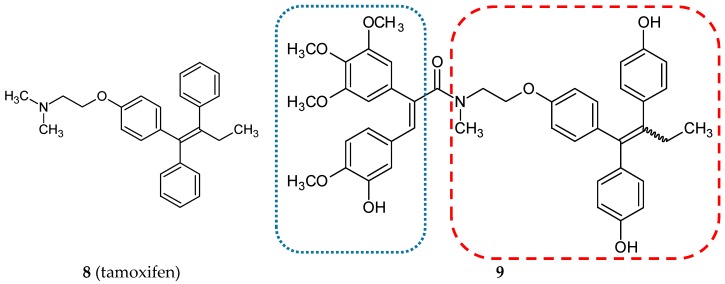
Chemical structures of tamoxifen (**8**) and its hybrid (**9**). Entity I (*cis*-stilbene) and entity II were depicted with the blue (**· · · · · · ·**), and red (**– – – –**) dotted lines, respectively.

In the treatment and prevention of estrogen-dependent breast cancers, selective estrogen receptor modulators are widely used. Tamoxifen (**8**) (Figure 4) is the most representative of this group of drugs. Its active metabolite, 4-hydroxy-N-desmethyl-tamoxifen, also referred to as endoxifen, was employed as a scaffold to create a new series of combretastatin hybrids [32]. All of the twenty amide-linked conjugates, CA-4, endoxifen, and hydroxyendoxifen were evaluated for antiproliferative activity against the estrogen receptor (ER)-positive MCF-7 breast cancer cell line. The compounds with a hydroxyl substituent in endoxifen pharmacophore exhibited a strong cytotoxicity to MCF-7 cells. The most active hybrid **9** (Figure 4 and Table 1) demonstrated a lower IC_50_ value than CA-4 (5 and 8 nM, respectively). Molecules selected on this basis were studied against the ER-negative MDA-MB-231 human breast cancer cell line. A reduction in antiproliferative activity in comparison to the ER-positive cell line was observed. These results proved that new conjugates are selective for the ER-expressing cell line. The most potent compound **9** was tested in the National Cancer Institute (NCI) using 60 cancer cell lines. The lead conjugate proved to be a strong growth inhibitor for cell lines such as RPMI-8226 (leukemia), NCI-H23 (non-small cell lung cancer), NCI-H460 (large cell lung cancer), HCT-116 (colon cancer), U251 (CNS cancer), MDA-MB-435, SK-MEL-2 (melanoma), OVCAR-8, SKOV-3 (ovarian cancer), ACHN, CAKI-1, RXF393, SN12C (renal cancer), MCF-7 (breast cancer), (GI%: 90.65, 96.76, 98.38, 97.57, 93.37, 90.98, 97.42, 97.21, 95.08, 96.43, 91.46, 95.02, 90.3, 94.3, respectively). Furthermore, the most probable explanation of the high specificity towards MCF-7 are the selective antagonistic effects on the over-expressed estrogen receptor. The effect of **9** and tamoxifen as a reference compound on alkaline phosphatase activity was tested using Ishikawa cells. This assay demonstrated that the lead hybrid does not exhibit estrogenic stimulation in the Ishikawa cell line, and in comparison to tamoxifen, it revealed reduced estrogenic stimulation over the investigated concentration range. Molecular docking studies visualized binding sites of compound **9** to estrogen receptors α and β [32].

#### 3.1.3. Nitrogen Mustard Hybrids

**Figure 5:**
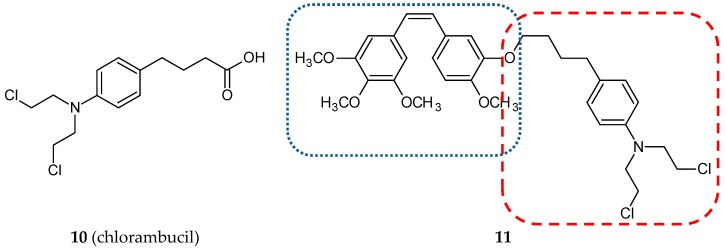
Chemical structures of chlorambucil (**10**) and its hybrid (**11**). Entity I (*cis*-stilbene) and entity II were depicted with the blue (**· · · · · · ·**), and red (**– – – –**) dotted lines, respectively.

Nitrogen mustards are nonspecific DNA alkylating agents. Since the early 1940s, when they were discovered as chemotherapeutic agents, numerous derivatives have been synthesized and evaluated. Their mechanism of action is based on the generation of an unstable aziridinum ion, which in the next step forms a monoalkylation adduct with DNA. As a result, cross-links between two complementary strands of DNA are formed, leading in consequence to cell death. Cyclophosphamide, chlorambucil (**10**) (Figure 5), melphalan, ifosfamide, and estramustine are most representative of this group of anticancer drugs [33]. Coggiola and her team published a study concerning combretastatin/nitrogen mustard hybrids [34]. They synthesized and evaluated three hybrids. Two were combretastatin A4 chimeras—a hydroxyl group of CA-4 joined via an ether linkage with 2-[bis(2-chloroethyl)amino]1-ethanol or with chlorambucil via an ester linkage. The third hybrid was made with the combretastatin A4 derivative—AC 7739 (a hydroxyl group substituted by an isosteric amino group). Cytotoxic studies were performed using the SH-SY5Y cell line (neuroblastoma). The hybrid compound of CA-4 with chlorambucil (**11**) (Figure 5) (Table 1) was significantly more cytotoxic than CA-4 (IC_50_ values: 0.64 vs. 1.5 nM). Chlorambucil presented a poor cytotoxic activity against the tested cell line. Inhibition of tubulin polymerization by synthesized chimeras was also confirmed in this study. 

#### 3.1.4. Pironetin Hybrids

**Figure 6:**
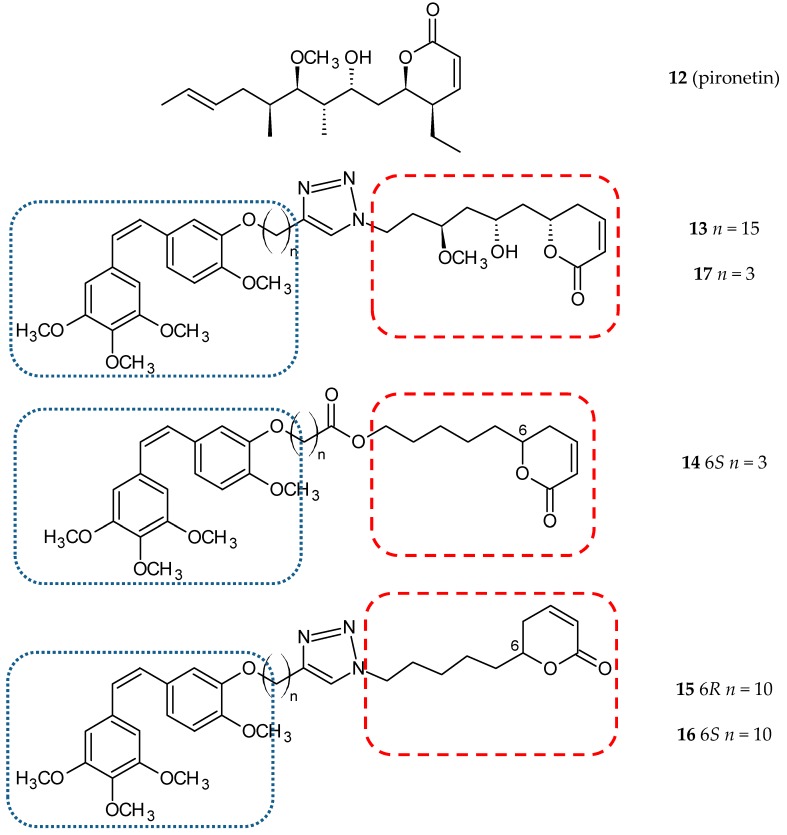
Chemical structures of pironetin (**12**) and its hybrid (**13-17**). Entity I (*cis*-stilbene) and entity II were depicted with the blue (**· · · · · · ·**), and red (**– – – –**) dotted lines, respectively.

Pironetin (5,6-dihydro-α-pyrone; **12**) (Figure 6) is a natural product isolated from the culture broth of *Streptomyces* sp. as a plant growth regulator [43]. Pironetin binds covalently with Cys316 of α-tubulin and impairs the conformation of the major loop and helix of α-tubulin to inhibit microtubule formation [44]. Torijano-Gutiérrez et al. synthesized a series of hybrid molecules of CA-4 and simplified pironetin linked by an ester-type spacer of variable length [45]. In further studies, they described the synthesis and evaluated the cytotoxic activity of the next series CA-4/pironetin hybrids fused by a 1,2,3-triazole spacer [35]. In both studies, the synthesized hybrids and their precursors were tested against two cancer cell lines, MCF-7 and HT-29, and one normal cell line, HEK-293. The authors calculated two coefficients, obtained by dividing IC_50_ values for normal cells by IC_50_ values for HT-29 (coefficient α) or by IC_50_ values for MCF-7 (coefficient β). Compound **13** (Figure 6) (Table 1) showed the best cytotoxic activity in the HT-29 cell line in comparison to CA-4 (0.40 ± 0.05 µM vs 4.2 ± 0.5 µM); coefficient α calculated for this molecule was 80, which means significant selectivity towards cancer cells (coefficient α for CA-4–5.9). The results of more detailed studies on the interaction mechanisms of the above hybrid compounds were published in 2015 [46]. The authors estimated the cytotoxic activity of ten new conjugate molecules, CA-4 and pironetin, as reference compounds. Hybrid molecules were selected on the basis of the previous study of the cytotoxic impact on the HT-29 cell line.

Those hybrids that did not induce the rounding-up of cells (expansion of mitotic cells) after incubation with the tested compound (100 µM) for one day were excluded. In this study, two human ovarian carcinoma cell lines, A2780 (sensitive to chemotherapy) and A2780AD (resistant to chemotherapy), were used. The highest cytotoxicity was observed for three compounds: **14**, **15**, and **16** (Figure 6). The resistance factor (RF—the ratio of the IC_50_ for A2780AD to IC_50_ for A2780) for most of the compounds under study was close to unity, which means that hybrid molecules of CA-4 and pironetin are also cytotoxic to multi-drug resistant A2780AD cells. It is suggested that the covalent binding of the studied hybrids with tubulin prevents the efflux pump from expelling the drugs out of cells. The effect of hybrid molecules of CA-4 and pironetin on the cell cycle was also evaluated in a non-small-cell lung adenocarcinoma cell line (A-549). CA-4 and pironetin arrested cells in the G2/M phase at the concentration of ≤ 0.05 µM. Among the compounds tested, two conjugates, **14** (containing ester-type linker) and **17** (Figure 6) (containing triazole linker), exerted the most potent antiproliferative effect against A-549. The same molecules displayed the strongest effect on depolymerization of the microtubule network in the immunofluorescence assay (25 and 50 µM, respectively; CA-4 and pironetin—0.05 µM). Critical concentration (CrC) values for the tested compounds ranged from 3.3–3.7 µM. Two of the known microtubule destabilizers, CA-4, and pironetin, presented CrC values higher than 6 µM in contrast to docetaxel (0.4 µM), the microtubule stabilizer. In the conclusion, the authors suggested that the type of linker and the length of the carbon chain can exert a certain, albeit not very significant, influence on cytotoxic activities [46].

#### 3.1.5. Benzoxazolone Hybrids

**Figure 7:**
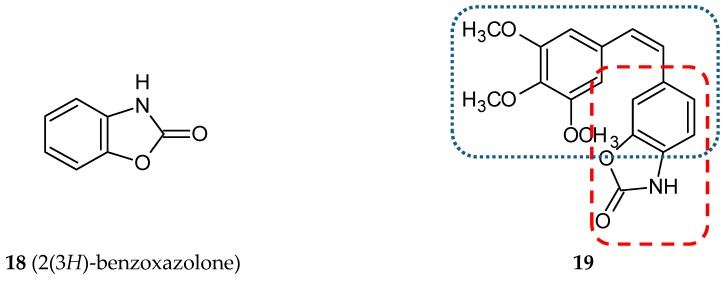
Chemical structures of 2(3*H*)benzoxazolone (**18**) and its hybrid (**19**). Entity I (*cis*-stilbene) and entity II were depicted with the blue (**· · · · · · ·**), and red (**– – – –**) dotted lines, respectively.

Mariana Gerova and her team designed and synthesized a new series of stilbene/benzoxazolone hybrids [47]. The stilbene B phenyl ring was replaced by a bioisosteric benzoxazolone heterocycle (bicyclic ring system composed of a phenyl ring fused to a carbamate) in *cis* and *trans* conformation. 2(3*H*)-Benzoxazolone (**18**) (Figure 7) derivatives are frequently used to modify a lead structure in the drug designing process [48]. Benzoxazolone derivatives, which exhibit numerous pharmacologic activities, are myorelaxant, sedative analgesic, antiseptic, anticonvulsant, and anti-inflammatory, antimicrobial, antifungal, anticholinergic, antipyretic, and anticancer agents [48,49,50]. All of the newly created hybrids composed of the A ring substituted with one, two or three methoxyl groups fused with benzoxazolone in the 4, 5, 6, or 7 positions via ethylene moiety in *cis* or *trans* configuration. Cytotoxicity tests were made for all twenty-eight new conjugates on three cell lines: HepG2 (human hepatocellular carcinoma cell line), EA.hy926 (human endothelial cell line) and K-562 (human chronic myelogenous leukemia cell line). A weaker cytotoxic activity of *trans*-stilbenes was observed in comparison to *cis*-isomers. The highest activity was presented by compound **19** (Figure 7) with the IC_50_ value ranging from 0.19–0.73 µM. Positional isomers of this hybrid demonstrated a loss of activity, which may suggest that crucial for their cytotoxic action is the position of the styryl fragment on the benzoxazole ring. The antiproliferative effects were evaluated for **19** and CA-4 as a reference compound on another eight cell lines, such as HT-29 (human colon adenocarcinoma), Colon-26 (mouse colon adenocarcinoma cell line), A-549 (human lung carcinoma cell line), MCF-7, MDA-MD-231 (human breast cancer cell lines), MCF-10A (human epithelial breast control cell line), HaCaT (human keratinocyte cell line) and NHEK (adult normal human epidermal keratinocytes cell line). Three of the cell lines used (A-549, MDA-MB-231, and HT-29) are relatively resistant to CA-4 treatment. In the case of three cell lines tested (HT-29, Colon-26, and A-549), **19** was more effective than a reference compound. Furthermore, **19** exhibited a lower toxicity than CA-4 against two normal cell lines. The effect of **19** on the cell cycle was determined using HepG2 cells. This study revealed the cell cycle arrest in the G2/M phase leading to apoptosis and cell death caused by a concentration lower than that for CA-4 [47].

#### 3.1.6. Benzothiazole Hybrids

**Figure 8:**
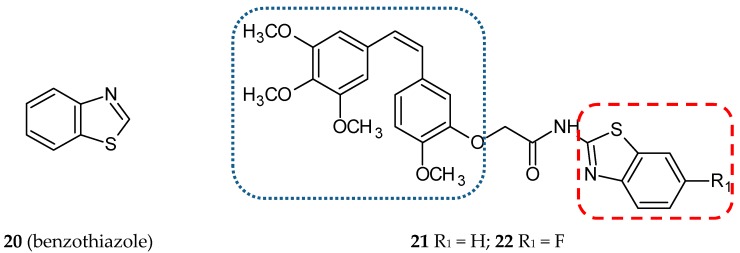
Chemical structures of benzothiazole (**20**) and its hybrids (**21, 22**). Entity I (*cis*-stilbene) and entity II were depicted with the blue (**· · · · · · ·**), and red (**– – – –**) dotted lines, respectively.

One of the important scaffolds of many biologically active compounds, including drugs, is a heterocyclic benzothiazole ring (**20**) (Figure 8). Among the many properties of this pharmacophore, those worth listing are antihelmintic, antimalarial, antitubercular, antidiabetic and antitumoral properties. The benzothiazole ring also occurs in drugs used in neurodegenerative diseases [51]. A new series of CA-4/amidobenzothiazole hybrids has been synthesized and described by Kamal et al., [36] who published a series of reports concerning hybrids with antitubulin activity. Two of them were focused on CA-4 conjugates. In this study, CA-4 and amidobenzothiazole were joined together with an amide bond formed with a hydroxyl group in the B phenyl ring of CA-4. The antiproliferative activity of synthesized compounds against seven cancer cell lines, such as A-549 (lung cancer cell line), Colo-205 (colon cancer cell line), THP-1 (leukemia cancer cell line), IMR-32 (neuroblastoma cell line), MCF-7 (breast cancer cell line), Hep-2 (liver cancer cell line), and PC-3 (prostate cancer cell line), was evaluated using the sulforhodamine B (SRB) method. The six most active hybrids were tested further using a panel of fifty-nine human cancer cell lines from the National Cancer Institute. The results obtained lead to the conclusion that anticancer activity depends on the nature of substituent linked to the benzothiazole part of the tested hybrids. The most promising hybrid, **21,** (Figure 8) (Table 1) exhibited the activity at the nanomolar level against twenty-three of the tested cell lines and sub-micromolar level activity against another twenty-two cell lines. Ten of the evaluated structures which showed a significant cytotoxic activity were analyzed in terms of their effect on cell viability (MTT assay) and the effect on the cell cycle (flow cytometry analysis). Also, **21** and **22** (Figure 8) turned out to be the most potent compounds with IC_50_ on MCF-7 at the 4 µM concentration and cell cycle arrest in the G2/M phase. The inhibitory effect on tubulin polymerization was observed only for **21**. The HTS tubulin polymerization assay revealed that **21** is a tubulin inhibitor of a potency comparable to CA-4. The immunofluorescence test proved that the tubulin network was disrupted by the hybrid tested. The influence of **21** and **22** on the mitogen-activated protein kinases (MAPKs) was investigated with Western blotting. This group of enzymes is involved in a variety of fundamental cellular processes, such as proliferation, differentiation, motility, stress response, apoptosis, and survival. The expression of ERK1/2 (extracellular signal-regulated kinases), transcription factor c-Jun (substrate for phosphorylation of c-Jun N-terminal kinases—JNKs) and phosphorylated ERK1/2 was evaluated. The levels of all the proteins quoted were decreased upon treatment with conjugates **21** and **22** in MCF-7 cells; the observed effect was more significant after treatment with **21**. Docking studies showed that these two congeners are able to bind in the ATP binding pocket of ERK protein; moreover, they fit well with the tubulin colchicine binding site [36].

#### 3.1.7. Cinnamic Acid Hybrids

**Figure 9:**
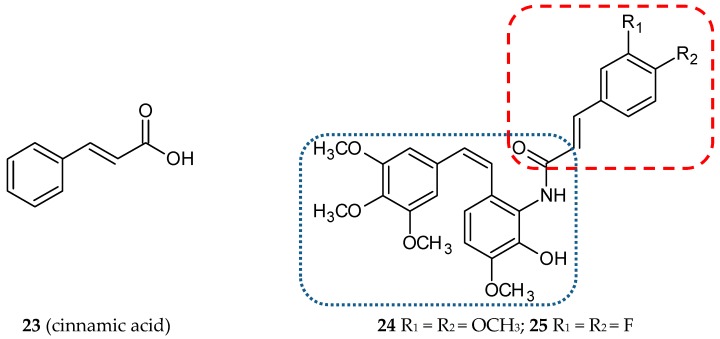
Chemical structures of cinnamic acid (**23**) and its hybrids (**24,25**). Entity I (*cis*-stilbene) and entity II were depicted with the blue (**· · · · · · ·**), and red (**– – – –**) dotted lines, respectively.

Many research groups are still looking for natural compounds exhibiting anticancer activities. In the last few years, cinnamyl compounds have been the focus of their attention. Cinnamic acid (**23**) (Figure 9) and its derivatives are natural compounds with high biological activities. *Cinnamomum cassia* Blume (Lauraceae), a plant cultivated in Asia, is a plentiful source of such compounds. These compounds display antioxidant, anti-inflammatory, antidiabetic, antifungal, and antitumor properties [52]. Kamal et al. decided to create new hybrids, conjugates of phenylcinnamide derivatives with amino-combretastatin analogs [37]. All synthesized compounds were evaluated for their cytotoxic potential against eight human cancer cell lines: MCF-7 (breast ER-positive), DU-145 (prostate), Hop-62 (lung), HeLa (cervical), K-562 (bone marrow), SK-OV-3 (ovary), Colo-205 (colon), and MIA-PaCa-2. CA-4 was used as a reference. The antiproliferative activities of the tested structures were the highest for MCF-7 and DU-145 cells. The most promising conjugates, **24** and **25,** (Figure 9) (Table 1) inhibited cell growth with GI_50_ values against MCF-7: 0.056 and 0.031 µM, respectively (CA-4 0.033 µM). They were further tested with respect to apoptosis induction, the effect on inhibition of tubulin polymerization, the loss of the mitochondrial membrane potential, and activation and cleavage of caspase-9 activity. Experimental data demonstrated that the cytotoxic activity of compounds **24** and **25** is a result of tubulin polymerization inhibition and subsequent apoptosis. The compounds described showed an arrest of the cell cycle in the G2/M phase, leading to caspase-dependent apoptotic cell death. The ability of **24** and **25** to inhibit tubulin polymerization was characterized by the IC_50_ values of 1.97 and 1.05 µM, respectively [37].

#### 3.1.8. Isocombretastatin Hybrids

**Figure 10:**
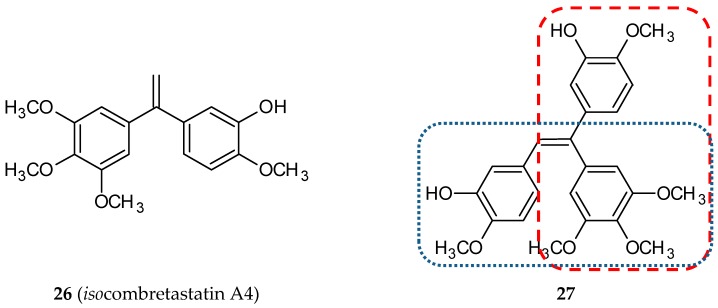
Chemical structures of *iso*combretastatin (**26**) and its hybrid (**27**). Entity I (*cis*-stilbene) and entity II were depicted with the blue (**· · · · · · ·**), and red (**– – – –**) dotted lines, respectively.

*Iso*combretastatins are a group of compounds containing a 1,1-diarylethylene scaffold. They are a third isomeric form in relation to *cis*-combretastatins and *trans*-combretastatins. They display potent anticancer activity and inhibit tubulin polymerization at the micromolar level [53,54]. This class of non-natural tubulin polymerization inhibitors is easier to synthesize because of the lack of need to control the olefin geometry. To combine the anticancer effects of CA-4 and *iso*CA-4 (**26**) (Figure 10), Rasolofonjatovo and her team synthesized and evaluated twelve new aryl olefins, hybrids of CA-4 and *iso*CA-4. [55]. The cytotoxic activity of these structures was tested against HCT-116 (human colon carcinoma cell line) with CA-4 and *iso*CA-4 used as reference compounds. Only three of the new derivatives had IC_50_ values lower than 10^-5^ M but still were not comparable with reference structures (both 2 × 10^−9^ M). For five of the tested compounds, micromolar values of IC_50_ for microtubule assembly were obtained. In summary, the results of the studies provided evidence that only compound **27** (Figure 10) has promising biological properties.

### 3.2. Cis-Restricted Stilbenoid-Based Hybrids

*Cis*-configuration of stilbene molecules is essential for their cytotoxic activities. To prevent *cis/trans* isomerization, *cis*-restricted combretastatins with a heterocyclic moiety in place of the ethene bridge were synthesized. These structures were further used in conjugations reactions to form hybrid molecules

**Figure 11:**
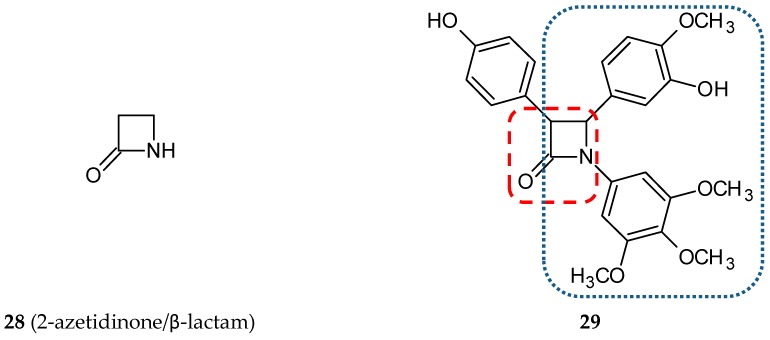
Chemical structures of 2-azetidione/β-lactam (**28**) and its hybrid (**29**). Entity I (*cis*-stilbene) and entity II were depicted with the blue (**· · · · · · ·**), and red (**– – – –**) dotted lines, respectively.

Another concept of hybrid formation is to join a molecule of the desired structure to the CA-4 ethylene bridge—in that way strongly cytotoxic CA-4-azetidinone hybrids were synthesized [38]. β-lactam scaffold (2-azetidinone, **28**) (Figure 11) can be found in many active compounds possessing anti-inflammatory, antibacterial, antiviral, and anticancer properties. A series of ten β-lactam *cis*-restricted molecules were synthesized and screened in three adenocarcinoma-derived colon cancer cell lines (CT-26, Caco-2, and the CA-4 resistant cell line HT-29). Notably, the structural modifications of the *cis*-restricted CA-4 molecule, namely the deletion or substitution of the meta hydroxy group with an amine conjugated amino acid and the introduction of an aromatic ring to β-lactam bridge, allowed it to overcome CA-4 resistance by enhancing the cytotoxicity of CA-4 hybrids 300-fold against HT-29 cells as compared to a parent compound. The lead hybrid (**29**) (Figure 11) exhibited improved chemical stability and strong cytotoxic activity against HT-29 cells in vitro and in the tumor xenograft model in vivo (Table 2) [38].

**Figure 12:**
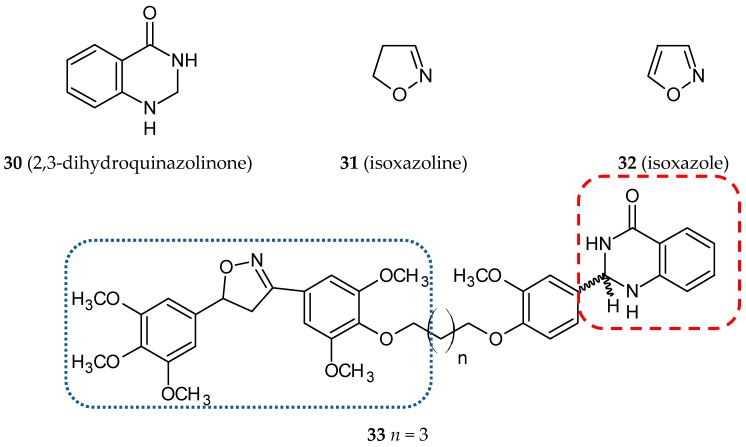
Chemical structures of 2,3-dihydroquinazolinone (**30**), isoxazoline (**31**), isoxazole (**32**) and their hybrid (**33**). Entity I (*cis*-stilbene) and entity II were depicted with the blue (**· · · · · · ·**), and red (**– – – –**) dotted lines, respectively.

Quinazolinones, N-containing heterocyclic compounds, naturally occur in plants and microorganisms. According to the positions of substituents linked to the quinazolinone scaffold, this group of pharmacologically-active molecules can be divided into four categories: 2-substituted quinazolinone, 3-substituted quinazolinone, 2,3-disubstituted quinazolinone, and quinazolinone derivatives. Biological properties depend strongly on the positions of the substituents. 2,3-dihydroquinazolinones (**30**) (Figure 12) demonstrates anticonvulsant, anticancer, antimicrobial and anti-inflammatory activities [56]. To realize the idea of hybrid molecules, Kamal et al. designed, synthesized, and evaluated a series of eight new 3,5-diaryl isoxazoline/isoxazole linked to 2,3-dihydroquinazolinone conjugates [57]. Isoxazoline (**31**) and isoxazole (**32**) (Figure 12) are five-membered nitrogen oxygen heterocycles that, on the one hand, perform many biological activities like other azoles and, on the other hand, may be used as stabilizers of a double-bond between the A and B rings in CA-4 analogs. The feature which differentiated the described hybrids, besides the type of azole ring, was the length of alkane spacers between pharmacophores. The most promising anticancer activity was observed for compound **33** (Figure 12). The cytotoxicity of this molecule was screened on a panel of sixty human cancer cell lines developed at the NCI. As a result of a 5-log dose range test, three dose-response parameters were calculated for all tested cell lines (GI_50_—the molar concentration required for half growth inhibition, TGI—the molar concentration leading to total growth inhibition, LC_50—_the molar concentration required for 50% cell death). Not only that, the authors also estimated the MG-MID (mean graph midpoint) for the listed parameters. This value characterizes an average activity for all cell lines. Hybrid **33** exhibited substantial anticancer activity against eighteen human cancer cell lines with GI_50_ values of less than 1 µM. The cytotoxic activity of the other seven hybrids was tested using five cancer cell lines (A-549, A2780, PC-3, MCF-7, KB) with the SRB method, as doxorubicin was used as a reference compound. The most sensitive cell lines were MCF-7 and PC-3. A cell viability (MTT) assay was accomplished for compound **33**, its conjugated moieties and CA-4 in MCF-7 cells. The tested hybrid exhibited amplified cytotoxicity compared to the parent constituents. In cells treated with **33**, the percentage of cells in the G2/M phase was increased compared to the control (DMSO) and conjugate partners. Furthermore, **33** was identified as an inhibitor of B1 and CDK1 (Western blot analysis) and also caused an increased level of cleaved PARP (Poly (ADP-ribose) polymerase, a family of related enzymes which play an important role in various cellular processes, e.g., DNA repair). In addition, the tested hybrid displayed a microtubule disruption effect along with fragmentation of nuclei at a level of concentration similar to that of CA-4 [57].

**Figure 13:**
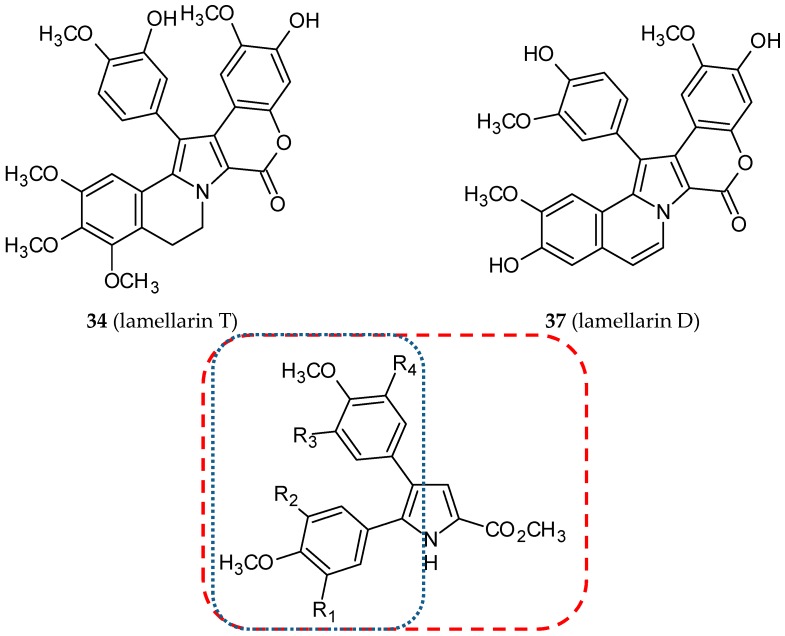

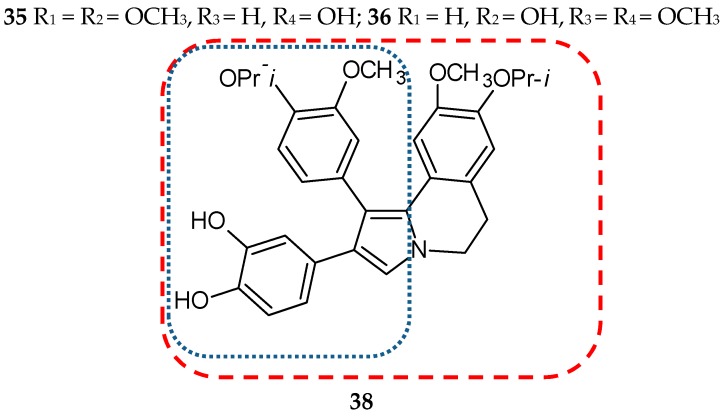
Chemical structures of lamellarins (**34, 37**) and their hybrids (**35, 36, 38**). Entity I (*cis*-stilbene) and entity II were depicted with the blue (**· · · · · · ·**), and red (**– – – –**) dotted lines, respectively.

Lamellarins are a family of pyrrole alkaloids naturally occurring in primitive marine animals such as mollusks, ascidians, and sponges. Since 1985, when the first lamellarin was isolated by Faulkner and his team, more than thirty different structures have been described. Most of them possess a pentacyclic 2-pyrrolo(dihydro)isoquinoline lactone core. The type of bond between C5-C6 and substituents linked to the rings differentiate these alkaloids. Lamellarins display numerous biological activities. Noteworthy among them are the inhibitory effects on cell division, cytotoxic activity, the inhibition of an HIV-I enzyme called integrase, and immune modulatory activity [58,59]. One of the representatives of this family of compounds is lamellarin T (**34**) (Figure 13). In 2006, Banwell and co-workers synthesized and evaluated six combretastatin A-4/lamellarin T hybrids [60]. 4,5-Diaryl-1*H*-pyrrole-2-carboxylate was established as a scaffold of designed molecules. All the molecules obtained have undergone biological studies, such as cytotoxic and mitotic index assays, tubulin assembly assays, and colchicine binding assays. Only two of the tested conjugates (**35** and **36**) (Figure 13) demonstrated an inhibitory effect similar to CA-4 on the binding of [^3^H]colchicine to bovine brain tubulin, indicating competition between colchicine and **35** and **36**. Compounds **35** and **36** were potent tubulin polymerization inhibitors with IC_50_ values of 1.4 and 1.3 µM, respectively (CA-4—1.1 µM). The growth of CA46 Burkitt lymphoma cells was inhibited by hybrids **35** and **36** with IC_50_ values of 29 and 31 nM, respectively (CA-4—3.2 nM). Among the synthesized compounds, one was significantly less active, whereas the other three hybrids were inactive. The authors suggested that the presence of N-bromosuccinimide in the pyrrole ring in three of the four inactive hybrids prevents the conformation of two aryl units required for antimitotic activity.

In 2010, twenty-six hybrids of CA-4 and lamellarin D (**37**) (Figure 13), which proved to be a potent pro-apoptotic agent, were synthesized and evaluated for their antiproliferative activity against five human cancer cell lines: K-562 (leukemia), A-549 (lung carcinoma), SMMC-7721 (hepatocellular carcinoma), SGC-7901 (gastric carcinoma) and HCT-116 (colon carcinoma) [61]. The studied hybrids were 1,2-diphenyl-5,6-dihydropyrrolo-[2,1-*α*]isoquinoline derivatives, designed as a result of a combination of structures of CA-4 and lamellarin D. Among the compounds tested, hybrid **38** (Figure 13), with two isopropoxy groups at positions 8 and 14 and two hydroxyl groups at positions 20 and 21, was the most active against all cancer cells studied.

**Figure 14:**
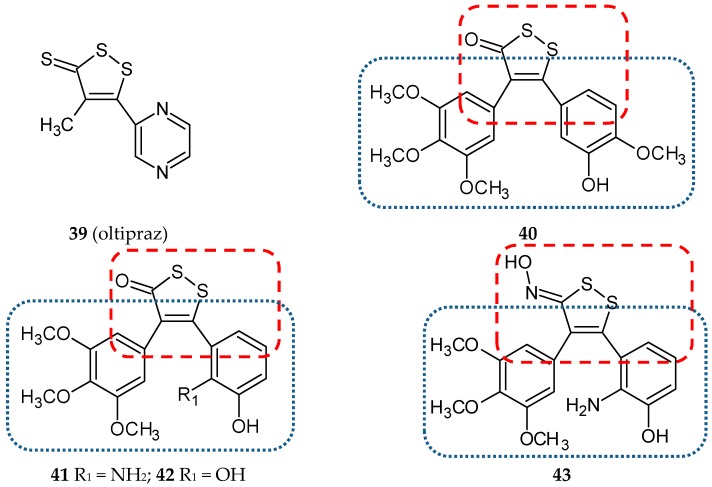
Chemical structures of oltipraz (**39**) and its hybrids (**40-43**). Entity I (*cis*-stilbene) and entity II were depicted with the blue (**· · · · · · ·**), and red (**– – – –**) dotted lines, respectively.

Oltipraz (4-methyl-5(pyrazinyl-2)-1-2-dithiole-3-thione, **39**) (Figure 14) was originally developed for the chemotherapy of a parasitic contagion called schistosomiasis. Further studies showed the effectiveness of oltipraz in chemoprevention of cancer of the skin, colon, lung, bladder, breast, liver, forestomach, and tracheal cancers. Furthermore, this 1,2-dithiolethione derivative possesses the ability to modulate liver regeneration and inhibit HBV and HIV replication. It is also a protector against hepatotoxicity caused by xenobiotics such as acetaminophen, carbon tetrachloride, and α-naphthylisothiocyanate [62]. The hybrid of oltipraz with CA-4, 5-(3-hydroxy-4-methoxyphenyl)-4-(3,4,5-trimethoxyphenyl)-3*H*-1,2-dithiol-3-one (**40**) (Figure 14) was synthesized, and its strong antitumor activity in hepatocellular carcinoma was demonstrated [63]. The reported structure was evaluated in many biological studies. Three hepatocellular carcinoma cell lines (HepG2, SMMC-7721, BEL-7402) and a normal liver cell line (HL-7702) were treated with different concentrations of **40**. The tested compound inhibited cancer cell-lines proliferation in a concentration-dependent way and showed less cytotoxic activity against normal liver cells. An analysis of the antitumor effect of **40** in vivo revealed reduced tumor growth in mice, comparable to CA-4 (inhibitory rates after dose 50 mg/kg: **40**—42.18%, CA-4—45.09%) (Table 2). The microtubule assembly assay confirmed that the tested conjugate inhibited the polymerization of microtubule in cell-free systems and disturbed the microtubule network in BEL-7402 cells. The cell cycle progression of the same hepatocellular carcinoma cell line after treatment with different doses of **40** was evaluated using flow cytometry and Western blot analysis. Taken together, both these tests suggested that the hybrid induces an arrest in mitosis, but not in the G2 phase. The authors indicated that mitotic slippage occurred after 36 h treatment with **40** because the levels of cyclin B1 significantly decreased and, moreover, the percentage of cells with multiple micronuclei gradually increased. In further experiments, the expression of the senescence-associated proteins (p14^Arf^, p53, Rb, β-actin, p-p53, p21, p16^INK4α^, p-Rb) was investigated. The obtained results showed that hybrid **38** promotes senescence in BEL-7402 cells via two classic tumor suppressor pathways: p14^Arf^-p53-p21and p16^INK4α^-Rb [63].

Two years later, Wang et al. designed and synthesized a new series of 4,5-diaryl-3*H*-1,2-dithiole-3-thiones and related molecules as *cis*-restricted CA-4/oltipraz hybrids [64]. The oltipraz core (1,2-dithiole-3-thione, -one or one oxime ring) was used to simulate a *cis* double bond in CA-4. The variability of the designed hybrids was also ensured with different substituents at the A or B phenyl rings of CA-4. The first step in the biological evaluation was an assessment of the effect of twenty-four synthesized hybrids on the cell viability of three human cancer cell lines: SGC-7901 (gastric carcinoma), KB (oral squamous epithelium carcinoma) and HT-1080 (fibrosarcoma carcinoma). Compound **41** (Figure 14) displayed the best potency against all tested cell lines with IC_50_ values of 0.172, 0.060, and 0.147 µM, respectively. For six of the newly-synthesized compounds, IC_50_ values were lower than 1 µM against at least two of the human cancer cell lines. Generally, the carbonyl derivatives were better cytotoxic agents than oxime and *O*-methylhydroxylamine analogs. As for thiocarbonyl derivatives, a lower antiproliferative potency was observed. Three conjugates, **41, 42,** and **43,** (Figure 14) were selected for further study. The compounds exhibited strong antitubulin activity leading to a cell cycle arrest in the G2/M phase and apoptotic death of cells. Confocal analysis of KB cells treated with **41** showed that this conjugate disrupted the structure of cellular microtubules. The studies were completed with molecular modeling which visualized the binding of **41** to the colchicine site located between the α and β subunits [64].

**Figure 15:**
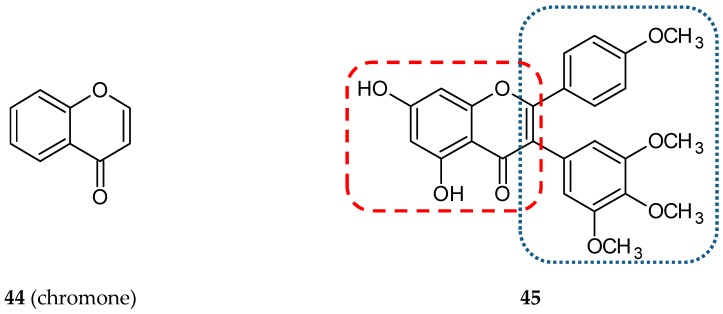
Chemical structures of chromone (**44**) and its hybrid (**45**). Entity I (*cis*-stilbene) and entity II were depicted with the blue (**· · · · · · ·**), and red (**– – – –**) dotted lines, respectively.

In a new series of combretastatin/flavone hybrids, the *cis* double bond was stabilized by chromone (**44**) (Figure 15) heterocycle [65]. Chromones are a family of bioactive compounds widely distributed in the natural world. They possess antiallergic, antimicrobial, antitumor, antidiabetic, and anti-inflammatory properties [66]. The position of the trimethoxyphenyl ring (C-2 or C-3 of the chromone) and the presence or absence of the C=O linker (C-3 of the chromone) were the differentiating features of new conjugates. The effect of the hybrids on tubulin polymerization was evaluated, and surprisingly, thirteen of the twenty hybrids were inactive. For the compounds which inhibited tubulin polymerization, the ratio of IC_50_ of the tested compound to IC_50_ of colchicine was calculated. This parameter, calculated for the most potent hybrid, **45** (Figure 15), deoxycombretastatin A4 derivative with the trimethoxyphenyl ring at C-2 of the chromone, was 2.4.

### 3.3. Phenstatin-Based Hybrids

**Figure 16:**
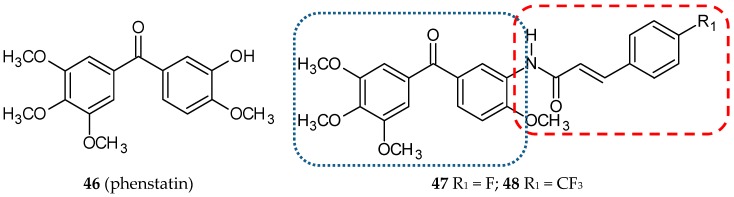
Chemical structures of phenstatin (**46**) and its hybrids (**47, 48**). Entity I (*cis*-stilbene) and entity II were depicted with the blue (**· · · · · · ·**), and red (**– – – –**) dotted lines, respectively.

Phenstatin (**46**) (Figure 16), a combretastatin A-4 benzophenone derivative in which a carbonyl group replaced the ethylene bridge, was firstly synthesized by Pettit et al. This modification resulted in an easier synthesis route because geometric selectivity is not required. Phenstatin is able to bind to the colchicine site of tubulin and demonstrates antineoplastic activity [67,68]. *Iso*combretastatins, reported earlier in this review, are compounds with both phenyl groups linked to carbon-1 of the ethylene bridge.

The first reports on this type of hybrids concern phenylcinnamide/phenstatin conjugates [69]. The cytotoxic activity of twenty-one new compounds were evaluated on seven cancer cell lines (HeLa, Me-180—cervical cancer cell lines, DU-145, PC-3—prostate cancer cell lines, Colo-205, HT-29—colon carcinoma cell lines, B-16—mouse melanoma carcinoma) with IC_50_ values ranging from 0.06–16.0µM. Hybrids with a fluoro group (**47**) (Figure 16) or trifluoromethyl substituent (**48**) (Figure 16) at the 4-position of the phenyl ring in the cinnamide moiety were the most potent cytotoxic agents. A flow cytometry analysis confirmed that these two compounds arrested the cell cycle in HeLa cells at the G2/M phase. Compounds **47** and **48** inhibited tubulin polymerization to an extent comparable to a phenstatin amino derivative used as a positive control, with IC_50_ values of 0.6, 0.7, and 0.6 µM, respectively. They induced the activation of capase-3, which, combined with the results of the DNA fragmentation assay and Hoechst staining (H33258), seems to suggest that hybrids **47** and **48** induce cell death by apoptosis. 

**Figure 17:**
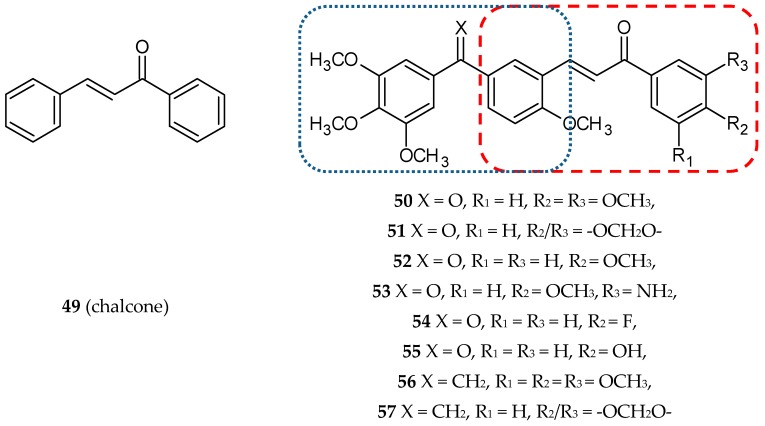
Chemical structures of chalcone (**47**) and its hybrids (**50–57**). Entity I (*cis*-stilbene) and entity II were depicted with the blue (**· · · · · · ·**), and red (**– – – –**) dotted lines, respectively.

Chalcones (benzylideneacetophenones or 1,3-diaryl-2-propen-1-ones, **49**) (Figure 17) are natural compounds occurring in fruits, vegetables, and other edible plants. They are precursors of flavonoids and isoflavonoids. Chalcones exhibit anti-inflammatory, antioxidant, antidiabetic, anti-obesity, antimicrobial, anticancer, and antileishmanial properties. They also exert a protective effect in fatty liver disease (alcoholic and nonalcoholic) and drug- and toxicant-induced liver damage [70]. The multiplicity of biological activities presented by chalcones were the rationale for designing the conjugates of chalcone with phenstatin or *iso*combretastatin [39]. A series of twenty-four new compounds were synthesized, and their antitubulin and pro-apoptotic properties were shown. Next, cytotoxic activity against two human breast cancer cell lines (MCF-7 and MDA-MB-231) was evaluated. All the tested structures exhibited a moderate to excellent cytotoxic effect with IC_50_ values in the range of 0.5–19.9 µM. Additionally, antiproliferative activities of the nine selected conjugates were tested against a panel of 60 human cancer cell lines at the NCI. Eight of the synthesized conjugates (**50–57**) (Figure 17) (Table 1) displayed significant antiproliferative activity against NCI-60 Human Tumor Cell Lines with the GI_50_ values ranging from 0.11–18.3 µM. Three structures (**50, 51, 53**) displayed the broadest spectrum of cytotoxic activity on the tested cell lines in the sub-micromolar range. For these compounds, more detailed tests were performed. The result of the flow cytometry revealed that the tested conjugates block the cell cycle at the G2/M phase and lead the cells to apoptotic death. Hoechst 33258 staining, caspase 9 activation, fragmentation of DNA, annexin V-FITC assay, and mitochondrial membrane depolarization assay confirmed the occurrence of apoptotic cell death. Three hybrids, **50, 51,** and **53,** inhibited the tubulin assembly with IC_50_ values ranging from 0.6–1.3 µM, as the corresponding values for phenstatin and CA-4 were 0.7 and 1.0 µM, respectively [39].

The studies of phenstatin and *iso*-phenstatin conjugates with fatty (oleic, linoleic and stearic) acids revealed their antiproliferative activity at the micromolar level. The antiproliferative activity of the studied conjugates increased with a degree of unsaturation of the fatty acid component [71].

### 3.4. Steroid-Based Hybrids

**Figure 18:**
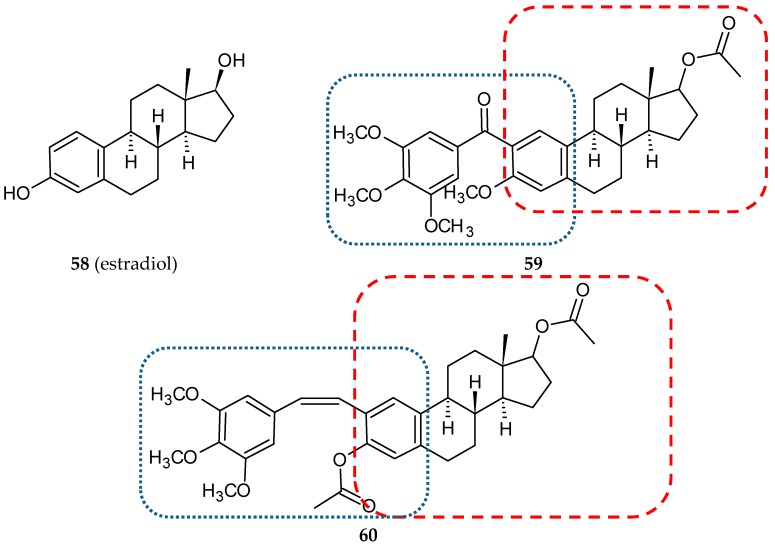
Chemical structures of estradiol (**58**) and its hybrids (**59, 60**). Entity I (*cis*-stilbene) and entity II were depicted with the blue (**· · · · · · ·**), and red (**– – – –**) dotted lines, respectively.

Hybrids of *cis*-stilbene analogs with steroids were designed as potential anti-breast cancer agents. Compounds that were combinations of phenstatin analogs and estradiol (**58**) (Figure 18) were synthesized, and their biological activities were evaluated by Parihar and co-workers [40]. To ensure the phenstatin configuration, the hybrids contained 3,4,5-trimethoxyphenyl as the A ring and the six-carbon aromatic ring of estradiol as the B ring. Cytotoxic activities of all fourteen hybrids against two breast cancer cell lines (ER-positive, MCF-7; ER-negative, MDA-MB-231) and a normal cell line(HEK-293, human embryonic kidney cells) were determined. Six of the studied compounds exhibited a significant activity (IC_50_ values lower than 15 µM) at least against one cancer cell line (two—MCF-7, four—MDA-MB-231). Almost all new conjugates were non-toxic against normal cells (IC_50_ values higher than 50 µM). Compound **59** (Figure 18) (Table 1), which was cytotoxic against hormone independent MDA-MB-231, exhibited antitubulin activity at a level comparable to podophyllotoxin. Estrogenicity and anti-estrogenicity were estimated in an in vivo test (Spargue-Dawley female rats) for all synthesized analogs. Estrogenic hybrids were not cytotoxic against MCF-7. Conjugate **59**, which exhibited a significant estrogen antagonistic activity and low estrogenicity, was selected for an in vivo acute oral toxicity study with the use of Swiss albino male and female mice (Table 2). After one oral dose of 300 mg/kg body weight, there were no significant changes in hematological and biochemical parameters such as total RBC, WBC count, hemoglobin, differential leukocyte count, serum total cholesterol, triglycerides, creatinine, ALT, and AST. Nor did the total body weight, absolute organ weight (lung, kidney, brain, spleen, heart), and relative organ weight (percentage of body weight) change significantly after a single dose treatment [40].

In another series of twenty-two conjugates, phenstatin pharmacophore was replaced with combretastatin A4 in both *cis* and *trans* conformation [72]. Among *cis* isomers, the most potent against breast cancer cell lines was compound **60** (Figure 18) with IC_50_ values against MCF-7 and MDA-MB-231 7.5 and 5.5 µM, respectively, lower than those for podophyllotoxin and tamoxifen. The antitubulin activity of this conjugate was very close to podophyllotoxin (IC_50_ values of 0.96 and 0.74 µM, respectively). A flow cytometry analysis after a 24-h treatment of 10 µM concentration of **60** and stained with propidium iodide demonstrated a cell cycle arrest in the G2 phase in MCF-7 cells. Furthermore, **60** led to significant cell membrane damage in tumor cells, which was observed through increased activity of lactate dehydrogenase (LDH). In studies of acute oral toxicity, the tested compound was non-toxic up to the dose of 300 mg/kg body weight (Table 2). In-silico docking of studied compounds to the colchicine site showed comparable values of binding energies for hybrid **60** and its precursors, CA-4 and podophyllotoxin. 

### 3.5. Other Related Hybrids

**Figure 19:**
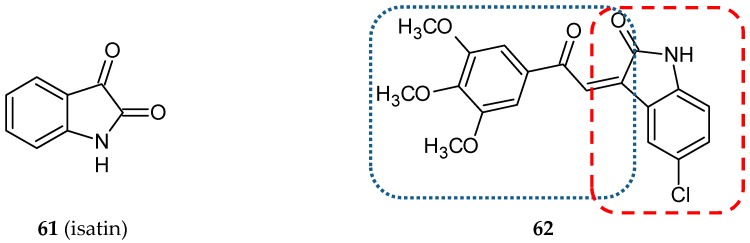
Chemical structures of isatin (**61**) and its hybrid (**62**). Entity I (*cis*-stilbene) and entity II were depicted with the blue (**· · · · · · ·**), and red (**– – – –**) dotted lines, respectively.

Isatin (1*H*-indole-2,3-dione, **61**) (Figure 19) is a heterocyclic molecule possessing an indol fragment. The structure is widely used in medicinal chemistry as a precursor of a great number of pharmacologically active compounds. Isatin and its derivatives are MAO inhibitors, muscle relaxants, immunosuppressants, and antimicrobial, antiviral, anticancer, antiallergic, anxiogenic and antithrombic agents [73]. To create a new multi-target anticancer compound, Cao and co-workers synthesized a hybrid compound named 3MCIC (**62**) (Figure 19) (3′,4′,5′-trimethoxy-5-chloro-isatinylchalcone), consisting of three entities derived from combretastatin, chalcone, and isatin [74]. The growth inhibitory effect of the obtained hybrid against five human cancer cell lines, HepG2 (human liver cancer cell line), MDA-MB-231, MCF-7 (breast cancer cell lines), CNE (nasopharyngeal carcinoma cell line), HCT-116 (colon cancer cell line) and L02 (human fetal liver cell line), was evaluated. It was found that **62** potently inhibited all the studied cancerous cell lines. However, the most sensitive to **62** treatment was HepG2 with the IC_50_ value of 4.5 µM. L02 used as a normal reference cell line was not affected by the tested hybrid. It is noteworthy that **62** appeared to be a more potent compound than sorafenib. The hybrid caused a rounding-up of HepG2 cells and massive vacuole accumulation in the cytoplasm. Downregulation of paxillin and focal adhesion plaques by **62** was responsible for the morphological changes of cells. Furthermore, the hybrid stabilized tubulin polymers in a way similar to paclitaxel. In summary, the novel hybrid influenced the essential factors involved in cell proliferation, cell cycle control, and the function of cytoskeleton proteins [74].

**Figure 20:**
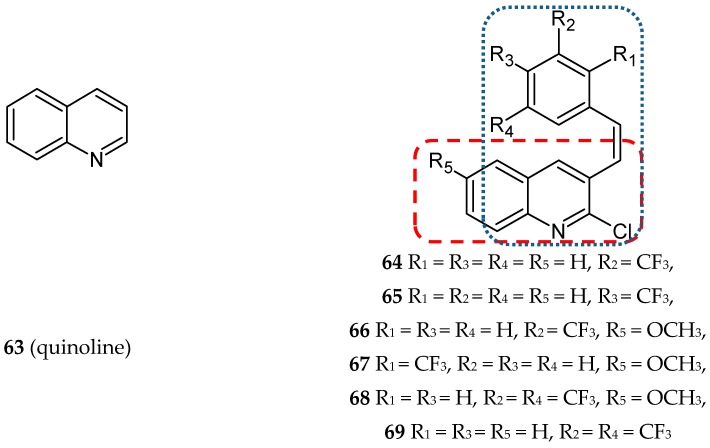
Chemical structures of quinoline (**63**) and its hybrids (**64**–**69**). Entity I (*cis*-stilbene) and entity II were depicted with the blue (**· · · · · · ·**), and red (**– – – –**) dotted lines, respectively.

The quinoline (other names: benzopyridine, benzo[b]pyridine, 1-benzazine, 1-aza-napthalene and benzazine, **63**) (Figure 20) scaffold is present in numerous pharmacologically-active compounds, both synthetic and naturally occurring. Many quinoline derivatives have been synthesized and described with regard to their properties. They have been found to exhibit antimicrobial, antimalarial, antifungal, anthelmintic, cardiotonic, anticonvulsant, anti-inflammatory, analgesic, and antitumor activities [75]. Srivastava and Lee synthesized a series of twenty-five novel conjugates of quinoline with *cis* or *trans* stilbene [76]. The antiproliferative activity of synthesized hybrids was tested using four cancer lines: HeLa (cervical carcinoma), MDA-MB-231 (ER-negative breast cancer), MCF-7 (ER-positive breast cancer), MDA-MB-468 (PTEN mutated breast cancer), and a non-cancer breast epithelial cell line 184B5. The studies revealed that *cis* derivatives were more cytotoxic and performed a better selective action against cancer cells than the compounds with *trans* configuration. Compound **64** was the most effective structure, although other *cis* derivatives—**65**–**68**, and **69** (Figure 20)—were also potent cytotoxic agents. Compounds **64**, **66** and **67** demonstrated prominent cancer cell growth inhibition (IC_50_ values in the range of 2.6–4.0 µM). Furthermore, they selectively inhibited the growth of cancer cells (with an IC_50_ value against a non-cancer cell line approximately two times higher). Structure-activity relationship studies revealed that the presence of the CF_3_ group linked in position 3 or 2 to the phenyl ring, occurring in compounds **64**–**67**, ensured a good cytotoxic activity. The influence of the most potent compound (**64**) on HeLa cells was tested using flow cytometry, Western blot analysis, and immunofluorescence microscopy. The results of these studies have shown that **63** arrests the cell cycle at the mitosis phase and can lead to apoptosis by impeding tubulin polymerization. The interaction of **64** with tubulin was studied using molecular modeling. The data from in silico studies suggest that **64** binds to tubulin at podophyllotoxin (PDT)-binding site [76]. 

**Figure 21:**
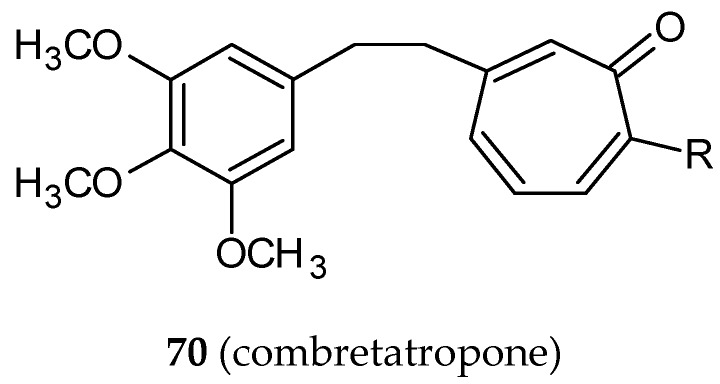
The chemical structure of combretatropone (**70**).

Combretatropones (a combination of a combretastatin and colchicine, **70**) (Figure 21) are hybrid molecules, first described in 1993 [77]. Structurally, they consist of two rings: A trimethoxyphenyl and a tropone. Tropones are a seven-carbon atom non-benzoic aromatic compounds with a carbonyl group. Plants and fungi are natural sources of troponoids: Tropones and their hydroxyl derivatives, tropolones. The troponoid moiety is a part of many biologically-active compounds [78,79]. In a study published in 2002, the authors described a synthesis of seventeen combretatropones derivatives differing from substituents on a tropone ring. Their ability to inhibit microtubule polymerization was evaluated [80]. The five most potent combretatropones were selected for further study. The inhibition of microtubule assembly in vitro by three five-member series of compounds conjugated with colchicine, combretatropones derivatives, and bicyclic colchicine C-10 substituted derivatives [81] was estimated. Colchicine analogs were the most potent antitubulin agents, while combretatropones were the least active among the compounds studied. In every group, the lowest IC_50_ value was achieved by a molecule with a –NHCH_3_ substituent. 

**Figure 22:**
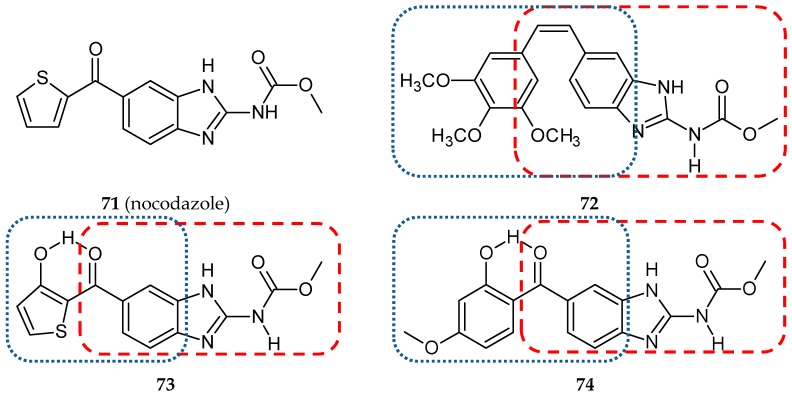
Chemical structures of nocodazole (**71**) and its hybrids (**72-74**). Entity I (*cis*-stilbene) and entity II were depicted with the blue (**· · · · · · ·**), and red (**– – – –**) dotted lines, respectively.

Conjugates with pyrazole and benzimidazole moieties effectively inhibited tubulin polymerization with IC_50_ at the micromolar level [82]. Nocodazole (**71**) (Figure 22), a member of the benzimidazole family, is a well-known inhibitor of microtubule polymerization that binds to the colchicine site. This depolymerizing agent arrests cell cycle progression at the G2/M phase [83]. Kale and co-workers designed and synthesized four conjugates consisting of two microtubule-interacting agents—nocodazole and a combretastatin [84]. A substituted phenyl ring of the combretastatin molecule replaced a thiophene ring in the nocodazole molecule. Hybrid **72** (Figure 22) was most similar to CA-4. All synthesized compounds were evaluated for their cytotoxic potential against the A-549 cell line. Only two hybrids (**72, 73**) (Figure 22) showed cytotoxic activity against A-549 cells with IC_50_ values of 12 and 6 µM, respectively, vs. the IC_50_ value of 0.28 µM for CA-4. Three synthesized compounds (**72–74**) (Figure 22) showed promising antitubulin activities with IC_50_ values of 1.6, 2.0, and 2.2 µM, respectively, but they were still less active than the CA-4 used as a positive control (IC_50_ value 1.1 µM). Additionally, the effect of the studied compounds on the binding of [^3^H]colchicine to tubulin was determined. The activities of these compounds as inhibitors of a colchicine binding to tubulin were also lower than those for CA-4, while the fourth synthesized compound appeared to be inactive. These observations were confirmed in the molecular docking studies. 

The studies on benzoselenazole-stilbene hybrids obtained by the combination of two pharmacophores, *trans*-resveratrol and ebselen (2-phenylbenzo[d] [1,2]selenazol-3(2*H*)-one), showing glutathione peroxidase(GPx)-like activity. Some of the synthesized hybrids exhibited antiproliferative activity against human cancer cell lines better than the parent compounds and inhibited thioredoxin reductase activity to an extent comparable to that shown by ebselen. The most promising hybrids contained a 3,4-dimethoxyphenyl or 3,4,5-trimethoxyphenyl ring and non-modified ebselen moiety [85].

## 4. Further Perspectives

**Figure 23:**
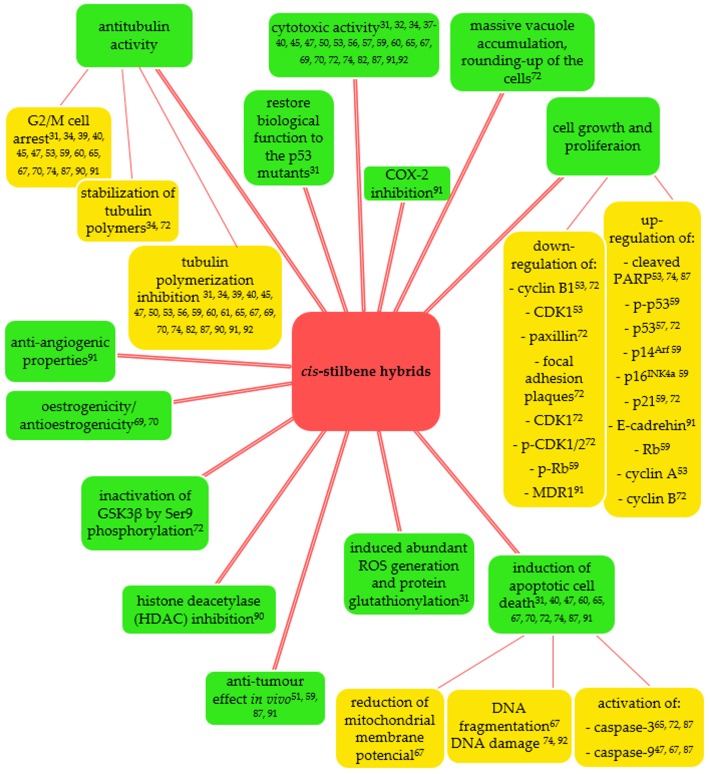
Biological activities of *cis*-stilbene hybrids.

A great number of newly synthesized hybrid compounds possessing a stilbene entity in their structure allowed us to select some of them which showed significant influence on cell growth and proliferation (Figure 23, Table 1). The dual action of hybrid molecules seems to promote the therapeutic efficiency of designed anticancer agents. However, only seven compounds were preliminary studied on animal models (Table 2).

The strategy to combine the *cis*-stilbene scaffold with the therapeutic agents of established activity appeared to be effective [31,32,33] and worth continuing. Recent reports by Kelly and co-workers concerned the design and synthesis of bifunctional compounds containing estrogen receptor (ER) ligands linked to tubulin-targeting CA-4. In the series of dual-action compounds, some hybrids incorporating endoxifen or cyclofenil as ER ligands exhibited high affinity to ERα and ERβ, and antiproliferation activity at a nanomolar level against human cancer cells [88]. The method of delivering the cytotoxic agent to tissue sites such as breast cancer using ER ligands conjugated with the antimitotic molecule of antiproliferation activity is worthy of development.

**Figure 24:**
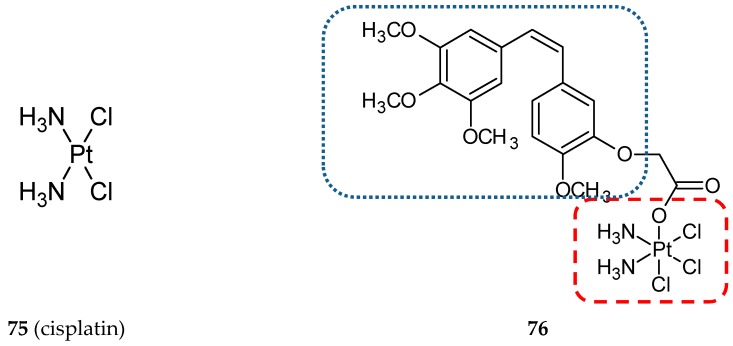
Chemical structures of cisplatin (**75**) and its hybrid (**76**). Entity I (*cis*-stilbene) and entity II were depicted with the blue (**· · · · · · ·**), and red (**– – – –**) dotted lines, respectively.

Cisplatin (**75**) (Figure 24) and its platinum(II)-based analogs are FDA-approved anticancer agents used in treatments of a variety of solid tumors [89]. Unfortunately, these DNA damaging drugs exhibit severe toxicity and side effects, which limit their use in therapy. To overcome the limitations of Pt(II)-based agents and multi-drug resistance of cancer cells, Pt(IV) hybrid prodrugs were designed with the use of CA-4. The experimental data confirmed better pharmacological and toxicological properties of Pt(IV) prodrugs containing active ligands in the axial position in relation to the cisplatin molecule [86,90]. A Pt(IV) combined with the CA-4 analog CA-platin (**76**) (Figure 24) exhibited potent cytotoxic activities against human cancer cell lines, including cisplatin-resistant cells. CA-platin significantly inhibited the tumor growth in the HepG2 xenograft model in vivo (Table 2); however, the hybrid was less effective than cisplatin given as a single agent and cisplatin given to mice simultaneously with CA-4 [86]. The authors supposed that the ether bond in the hybrid molecule is too strong to be cleaved, and as a result, CA-4 is not efficiently released in the body. On the other hand, the cleavage of the hybrid does not seem to be essential for its anticancer activity. Further studies on hybrid bioavailability would be required.

Another anticancer therapeutic agent, doxorubicin, was conjugated with CA-4 via a photoremovable protecting group to form a photoresponsive hybrid prodrug [91]. Obtained hybrid molecules exhibited increased cytotoxicity against MDA-MB-231 cells compared with individual drugs. 

**Figure 25:**
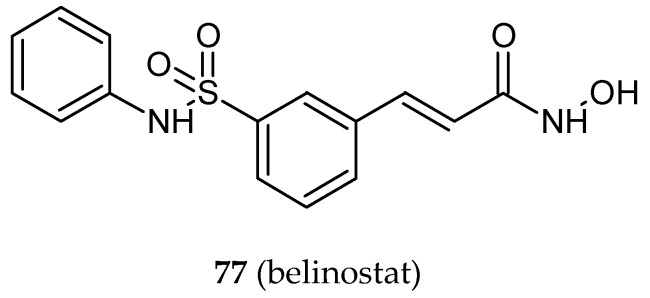

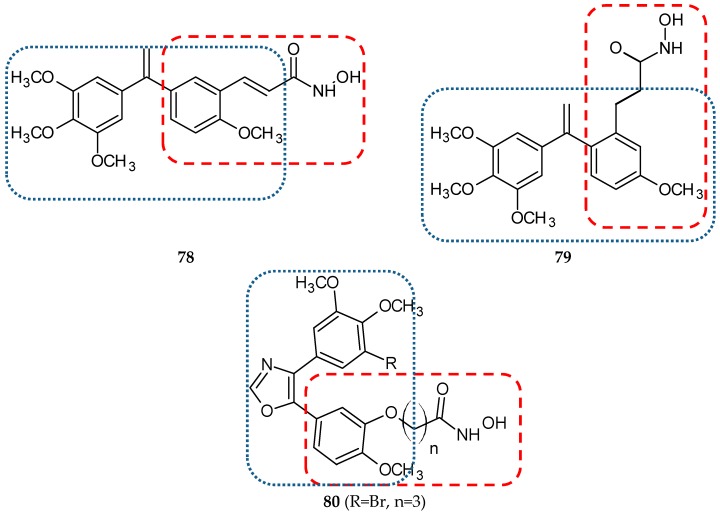
Chemical structures of belinostat (**77**), it’s hybrids (**78, 79**, **80**). Entity I (*cis*-stilbene) and entity II were depicted with the blue (**· · · · · · ·**), and red (**– – – –**) dotted lines, respectively.

The overexpression of histone deacetylases (HDACs) occurs in various types of human cancers. Thus, these enzymes are targets of anticancer treatment. A series of hybrid molecules of 1,1-diarylethylenes (*iso*CA-4) and belinostat (**77**) (Figure 25), an HDAC inhibitor and a drug approved in hematological malignancies and solid tumors, was designed and synthesized. Compounds **78** and **79** (Figure 25) (Table 1) exhibited strong antiproliferative activity arisen from the inhibitory effect on tubulin polymerization and the inhibition of HDAC8 activity [41]. In the valuable studies of Schmitt and co-workers [87], oxazole-bridged CA-4 was attached to alkyl tethered hydroxamic acids of varying length as HDAC inhibitors (**80**) (Figure 25). The antiproliferative effect and antitubulin activity were the most pronounced for derivatives with 4- and 5-atom spacers, whereas HDAC inhibition increased for those with longer spacers. Notably, the hybrids exhibited significant selectivity for cancer cells over non-malignant cells.

**Figure 26:**
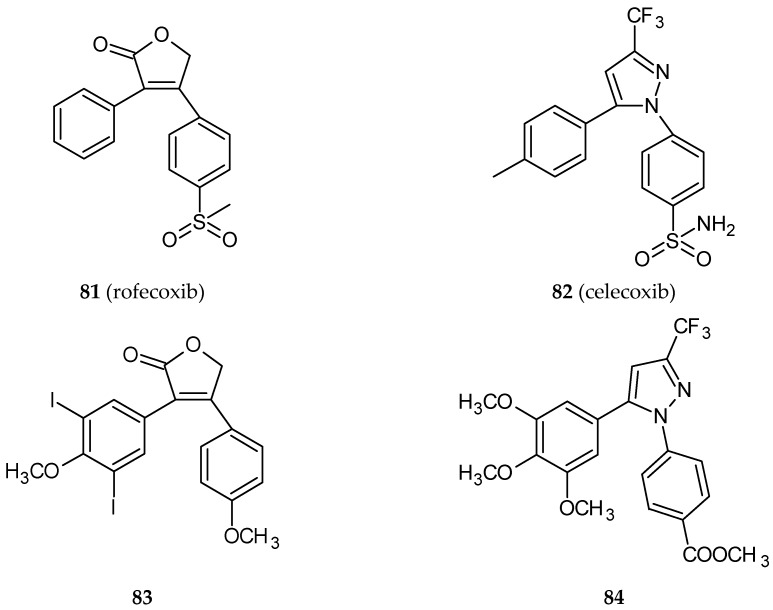
Chemical structures of rofecoxib (**81**), celecoxib (**82**) and their hybrids (**83, 84**).

Recent papers concern a synthesis and biological evaluation of analogs of *cis*-restricted CA-4, which has a structure similar to known cyclooxygenase-2 inhibitors, rofecoxib (**81**) (Figure 26) and celecoxib (**82**) (Figure 26) [42,92]. Cyclooxygenases (COXs) are a family of enzymes comprising three isoforms involved in the first two steps of prostanoid biosynthesis. Upregulation of isoform 2 (COX-2) is a result of the action of proinflammatory agents. There are known COX-2 inhibitors used as nonsteroidal anti-inflammatory drugs (NSAID) named coxibs, which are tricyclic *cis*-stilbene derivatives. One of the first developed drugs belonging to this group of pharmaceuticals was rofecoxib. In 2004, rofecoxib was withdrawn from the market due to its cardiotoxicity. Because of COX-2 overexpression in some types of cancers, COX-2 inhibitors such as rofecoxib can find a new application [93]. The cytotoxic activity of the synthesized hybrid molecule **83** (Figure 26) (Table 1) was evaluated against four human colon cancer cell lines: HT-29, HCT-116, SW620, LoVo. The HT-29 resistant to CA-4 cell line was very sensitive to the newly tested compound with an IC_50_ value nearly 20-fold lower in comparison to CA-4. Cell-free tubulin polymerization assay and tests using cancer cell lines (CA-4 sensitive and resistant) confirmed that compound **83** inhibits tubulin polymerization. Furthermore, the hybrid caused a decreased level of overexpressed COX-2 protein in colon cancer cells. An inhibitory effect of **83** on the invasion and migration of colon cancer cells was also assessed. What is more, the anti-angiogenic properties of **83** were proven by an inhibitory effect on HUVEC (human umbilical vein endothelial cells) migration. The drug caused also a concentration-dependent decrease in the total length of capillary tubes formed by HUVE cells. The flow cytometry analysis proved a cell cycle arrest in the G2/M phase and an induction of apoptosis by upregulation of apoptotic protein BAX and PUMA, cleaved caspase-3, and cleaved PARP as a result of the treatment of cells with **83**. These observations were confirmed in in vivo tests using a mouse xenograft model (Table 2). After a single dose of 25 mg/kg, no organ toxicities were evident. Molecular docking studies suggest a dual mode of action of the conjugate—it can be bound to the colchicine binding site of tubulin and to the COX-2 active site [42].

Combretastatin-(trifluoromethyl)pyrazole analog (**84**) (Figure 26) is a hybrid molecule that resembles another cyclooxygenase-2 inhibitor, celecoxib. The compound exhibited pronounced cytotoxicity against HeLa, B16F10, and multidrug-resistant mammary tumor cells EMT6/AR1. It depolymerized the interphase microtubule and arrested MCF-7 cells at mitosis. It inhibited the assembly of tubulin in vitro interacting with tubulin at the colchicine binding site [92]. 

## 5. Summary

The design of multifunctional compounds is a relatively new concept in drug development, which has its origins at the end of the twentieth century. This strategy establishes the design of hybrid compounds made of two or more biologically-active pharmacophores. Molecules obtained in this way may exert synergistic effects and possess better pharmacokinetics properties or higher bioavailability. Another goal of this strategy is to minimize potential drug resistance and drug-drug interactions. In this review, we have surveyed the reports published in the last few years, concerning a group of hybrid molecules containing *cis*-stilbene moiety. The compounds discussed in the review show multifunctional biological activities as a result of their complex chemical structures. The compounds that deserve special attention are **9**, **11**, **13**, **21**, **25**, **53**, **59**, **78** and **83**. These molecules possess higher antiproliferative activities against some cancer cell lines than the most representative combretastatin family member, i.e., CA-4 (Table 1). Moreover, some of the described hybrids demonstrating prominent cytotoxicity—**19**, **25**, **51**, **53**, **59**, and **83**—are as efficient as tubulin polymerization inhibitors as CA-4. Many previous studies concerning CA-4 analogs suggested that the presence of the 3,4,5-trimethoxyphenyl entity occurring in the CA-4 molecule is indispensable to the process of tubulin polymerization inhibition. In fact, this pharmacophore is an attribute characteristic of all of the most potent molecules, except compound **83**, which has methoxy groups substituted with iodine atoms.

To summarize, data from experimental studies suggest that the idea of *cis*-stilbene hybrids may provide a useful tool in seeking efficacious anticancer agents. New perspectives in designs of novel multi-target agents against cancer may be developed on the basis of the recent studies of CA-4 hybrids with therapeutic agents, i.e., cyclooxygenase-2 inhibitors, ER ligands, HPAC inhibitors, and anticancer medications, cisplatin, and doxorubicin. However, for the time being, only some of the hybrid compounds are in phases of preclinical study and, to the best of our knowledge, none of the hybrids described in this review have entered clinical trials. Further pharmacologic studies of the most promising hybrid compounds would be recommended.

## Figures and Tables

**Table 1:** Cytotoxic activity of selected hybrid molecules.

Hybrid Molecule	Entity I	Entity II	Cell Line	Ratio #	Ref.
9	Tamoxifen	CA-4	MCF-7	0.625 *^a^	[32]
11	Chlorambucil	CA-4	SH-SY5Y	0.427 *^a^	[34]
13	Pironetin	CA-4	HT-29	0.095 *^a^	[35]
21	Amidobenzothiazole	CA-4	EKVX	0.481 **^a^	[36]
25	Phenylcinnamide	CA-4	MCF-7	0.939 **^a^	[37]
			DU145	0.978 ^**a^	
29	2-Azetidinone	CA-4	CT-26	0.746 *^a^	[38]
			Caco-2	0.371 *^a^	
			HT-29	0.006 *^a^	
53	Chalcone	Phenstatin	MCF-7	0.625 *^b^	[39]
			MDA-MB-231	0.800 *^b^	
59	Estradiol	Phenstatin	MDA-MB-231	0.714*^c^	[40]
78	Belinostat	*iso*CA-4	HCT-116	0.750 **^d^	[41]
83	Rofecoxib	CA-4	HT-29	0.058 *^a^	[42]
			LoVo	0.676 *^a^	

# Ratio was calculated by dividing IC_50_ * or GI_50_ ** of hybrid molecule by IC_50_ * or GI_50_ ** of reference standard. ^a^ combretastatin A-4, ^b^ phenstatin, ^c^ tamoxifen, ^d^ *iso*combretastatin A-4 were used as reference.

**Table 2:** In vivo studies involving hybrid molecules.

Hybrid Number	Dose	Model (Animal/Sex/Tumor Cell Line)	Main Outcomes (Cancer)	Pharmacological Effects/Toxicity/Adverse Effects	Ref.
**28**	40 mg/kg (i.p.) single dose	Tumor xenograft (Mi/F/CT-26)	decrease tumor volume after 11 days	rough coat, diarrhoea, loss of appetite, mortality rate 1/5	[38]
**40**	25, 50, 100 mg/kg/2 days (i.p.)	Tumor xenograft (Mi/M/BEL-7402)	significantly lower size and weight of the tumors	no differences observed in body weight, heart, liver, spleen, lung, kidney, brain	[63]
**59**	5, 50, 300 mg/kg (p.o.) single dose	(Mi/F and M)	−	no significant changes in tested haematological and biochemical parameters	[40]
**60**	5, 50, 300, 1000 mg/kg (p.o.) single dose	(Mi/F and M)	−	no significant changes in tested haematological and biochemical parameters up to dose 300 mg/kg	[72]
**76**	5, 10 mg/kg/week (i.v.)	Tumor xenograft (Mi/F/HepG-2)	high antitumor activity (better than CA-4)	lower body weight loss in comparison to cisplatin	[86]
**80**	1 × 100 mg/kg (i.p.) 1 × 200 mg/kg (p.o.)	(Nude Mice)	−	no significant toxicity (no weight loss, normal behaviour)	[87]
**83**	25 mg/kg/day (i.p.)	Tumor xenograft (Mi/F/HT-29)	reduction of the overall tumor weight compared to CA-4 treatment	no remarkable changes in the average body weights; no significant changes in liver, kidney, heart, spleen, and brain tissues	[42]

i.p.—intraperitoneal, i.v. —intravenous, p.o—per oral, Mi—mice, F—female, M—male, CT-26—colon carcinoma, BEL-7402—hepatocellular carcinoma, HepG-2—hepatocellular carcinoma, HT-29—colon adenocarcinoma, CA-4—combretastatin A4.
